# Schizophrenia and toxoplasmosis: association with catatonic symptoms

**DOI:** 10.17650/2712-7672-2020-1-1-22-29

**Published:** 2020-09-02

**Authors:** Dmitry V. Romanov, Aleksey Iu. Brazhnikov, Denis S. Andreyuk, Natalia V. Zakharova, Lidia V. Bravve, Vasilisa A. Kovaleva, Evgeniya V. Abbazova, Dmitriy B. Goncharov, Irina V. Titova, Elvira A. Domonova, George P. Kostyuk

**Affiliations:** I.M. Sechenov First Moscow State Medical University (Sechenov University); Mental Health Research Centre; Mental-health clinic No. 1 named after N.A. Aleхeev; M.V. Lomonosov Moscow State University, Faculty of Economics; N.F. Gamaleya Federal Research Center for Epidemiology and Microbiology, Ministry of Health of Russia

**Keywords:** schizophrenia, toxoplasmosis, T.gondii, catatonia, Positive and Negative Syndrome Scale, PANSS, шизофрения, токсоплазмоз, T.gondii, кататония, шкала позитивных и негативных синдромов, PANSS

## Abstract

**Introduction.:**

The association between schizophrenia and toxoplasmosis has been demonstrated in a number of studies: the prevalence of schizophrenia is significantly higher in toxoplasmosis positive subjects than in those with *T. gondii* negative status. However, the clinical significance of this association remains poorly understood.

**Objectives.:**

To identify clinical phenomena that are typical for toxoplasmosis-associated (*T. gondii* seropositive) schizophrenia compared to Toxoplasma-seronegative schizophrenia.

**Methods.:**

A retrospective database analysis of serum samples from 105 inpatients with schizophrenia (ICD-10code: F20; including 55 male patients; mean age of 27.4 6.4 years) was carried out. The clinical examination involved a structured interview including ICD-10 and E. Bleulers criteria for schizophrenia and psychometric tests(Positive and Negative Scales of PANSS). Serum antibodies (IgG) to *T. gondii* were identified using ELISA. The statistical significance of any differences were evaluated using the non-parametric Mann-Whitney (U) and X^2^ tests.

**Results.:**

The proportion of seropositive patients in the sample was 16.2%. Comparing schizophrenia patients, who were seropositive or seronegative for toxoplasmosis, there were no statistically significant differences for the mean total PANSS score, mean PANSS-P, PANSS-N or PANSS-G scores. For the majority of PANSS items, differences were also statistically insignificant, except for G5 and G6mannerism and posturing. Seropositive patients had a higher score for this item than seronegative patients: 3.5 versus 2.1 points (U=389.5; р=0.001). Depression, on the contrary,was less pronounced in seropositive than seronegative patients: 1.4 versus 2.4 points (U=509.5; р=0.023). In addition,in seropositive patients, the frequency of symptoms such as mutism according to ICD-10 criteria for schizophrenia was significantly higher (23.5% versus 3.4%, X^2^=9.27, р=0.013), and the whole group of catatonic symptoms according to the E. Bleulers criteria for schizophrenia was higher (52.9% versus 28.4%, X^2^=3.916, p = 0.048).

**Conclusion.:**

The association between a positive toxoplasmosis status in patients with schizophrenia and catatonic symptoms has been revealed for the first time and should be verified in larger studies.

## INTRODUCTION

A significant association between schizophrenia and toxoplasmosis caused by the neurotropic pathogen of *T. gondii* has been identified in a number of clinical and epidemiological studies, the results of which are illustrated, for example, in the meta-analysis by J. Gutierrez-Fernandez et al. [1]. The latter demonstrates a statistically significant correlation between the *T. gondii*-positive status and schizophrenia with the odds ratio (OR) of 2.50 (95% CI=1.40–4.47). This means that following *T. gondii* infection, the probability of schizophrenia diagnosis is 2.5-fold higher compared to patients with *T. gondii*-negative status. In general, the OR ranges from 1.47 (95% 0=1.03-2.09) to 2.73 (95% 0=2.10-3.60) in meta-analyses and some large epidemiological studies [2-4].

Data on the prevalence of infection in patients with schizophrenia (seropositive for IgG) also vary across a fairly wide range: from 16.2% to 46% [5,6].

According to a study of the Russian population of schizophrenia patients, the prevalence of the infection is as high as 40%: 62 of 155 schizophrenia patients were seropositive for toxoplasmosis (IgG) versus 39 of 152 healthy control group subjects (25%); the odds ratio being 1.93 [7].

The clinical significance of seropositive *T. gondii* status in schizophrenia patients remains poorly understood. Similarly, knowledge of the possible correlates at the symptomatic, syndromal and dimensional levels is sparse and available data largely controversial.

A comparison of Toxoplasma-seropositive patients with schizophrenia and seronegative patients has been carried out in only a few studies. F. Dickerson et al. [5] included 358 patients with schizophrenia in their study and found that seropositive patients differed significantly from seronegative patients by gender only (most patients were female, p=0.021). However, there were no significant differences in respect of age, race, level of education, age at onset, duration of schizophrenia, or for total PANSS or Repeatable Battery for the Assessment of Neuropsychological Status (RBANS) scores, smoker status, prevalence of diabetes or the use of antipsychotic drugs.

T. Çelik et al. [6] showed in a sample of 90 patients with schizophrenia that only a continuous disease course was associated with a seropositive status. No similar relationship was observed for parameters such as lack of insight or type of schizophrenia according to ICD-10 (paranoid, undifferentiated, residual, disorganized, catatonic).

A small number of scientific papers present the results of clinical and laboratory associations of toxoplasmosis in schizophrenia with individual dimensions of the mental illness. G.P. Amminger et al. [8] showed that a higher plasma level of IgG was significantly associated with a greater severity of positive symptoms on the Brief Psychiatric Rating Scale (BPRS) in a group of patients with an ultra-high risk of psychosis (attenuated or short-term psychotic symptoms).

In a study by D. Holub et al. [9], seropositive schizophrenia patients demonstrated statistically significant differences in the severity of symptoms (p=0.032) assessed using the PANSS-P (Positive Symptoms Subscale) but not PANSS-N (Negative Symptoms Subcale) or General Psychopathology PANSS-G subscale, when compared to seronegative subjects. The authors noted that the onset of schizophrenia in infected male patients occurred approximately a year earlier than in non-infected males while in infected female patients it was three years later than in non-infected female patients. In addition, the last hospital stay at the time of the study was, on average, 33 days longer than in the case of seropositive schizophrenia patients than in seronegative patients (p=0.003).

Comparable data were obtained in one of the largest studies on the association between schizophrenia and toxoplasmosis, which included 600 patients with the first episode of schizophrenia [10]. The authors showed that there were no significant differences between the groups in terms of parameters such as gender, age, level of education, disease duration, age at the time of schizophrenia onset, living conditions (urban, rural) or family history of mental illness. They also demonstrated that schizophrenia patients with chronic toxoplasmosis had a higher PANSS Positive Subcale score (mean PANSS-P score: 20.8 versus 19.4), cognitive symptoms score (mean score: 8.5 versus 7.7) and excitement score (mean score: 9.5 versus 7.9) and a significantly lower PANSS Negative Subcale Score (mean PANSS-N score: 16.4 versus 17.8) compared to seronegative patients. However, none of the above publications provides an analysis of differences in individual PANSS scale items or schizophrenia symptoms.

Therefore, the goal of the study was to identify clinical phenomena that are typical for toxoplasmosis-associated (*T. gondii* seropositive) schizophrenia compared to seronegative schizophrenia.

## MATERIALS AND METHODS

### Study Design 

A retrospective database analysis of serum samples obtained from schizophrenia patients was carried out. The patients were included in the databases as part of the research programme "International approaches to studying human and society mental health" conducted in the Mental-health clinic No. 1, named after N.A. Alexeev since 2017. The study was approved by the independent interdisciplinary ethics committee of clinical studies on 14thjuly, 2017(meeting minutes No. 12). Serum samples obtained from schizophrenia patients were collected from January 2019 to February 2020.

**Inclusion Criteria** included schizophrenia diagnosis according to the ICD-10 criteria (F20); age >18 years and signature on the informed consent form for both participation in the study and testing of biological samples (blood serum).

**Exclusion Criteria** included structural CNS disorders (F0) according to the ICD-10 criteria, psychoactive substance dependence (F1), affective psychoses (F3) according to the ICD-10 criteria and exacerbations of autoimmune disorders that could potentially distort the results of serology testing.

Clinical examination of patients was carried out in the first days of their hospital stay by two psychiatrists using a single structured interview and also collecting social, demographic and certain clinical symptoms of schizophrenia. The interview included the criteria for schizophrenia diagnosis according to the ICD-10 (important symptoms, some of which are observed during most of an episode lasting one month or longer) supplemented by E. Bleuler's criteria [11] and a psychometric evaluation of symptoms using the PANSS scale [12,13].

**Blood sampling** was performed once from the cubital vein in the morning under fasting conditions between 8.00 and 8.30 am. Blood samples were placed in test tubes with ethylenediaminetetraacetic acid (EDTA) and transported to the laboratory within two hours to material safety requirements. Blood was centrifuged at 750 g for 15 min at 22°C; plasma was collected and subsequently used for testing. Frozen samples were stored at -18° to -24°C.

**Serology Testing Methods.** Serum levels of anti-*T. gondii* antibodies (IgG) were determined in the blood samples using ELISA and a ToxaplaStripG kit (Certificate 9398-005-4037-1634-2008).

**Statistical Analysis Methods.** Differences in quantitative ordinal variables (mean total PANSS scores, PANSS-P score, PANSS-N score, PANSS-G score, as well as individual PANSS items) were evaluated using the Mann-Whitney test (U). The nonparametric chi-squared test (χ^2^) was used to compare the frequencies of categorical variables (individual symptoms of schizophrenia according to the ICD-10 and the E. Bleuler's criteria [11]). The level of statistical significance was 0.05.

**Sample Characteristics.** The study included 105 patients with schizophrenia (including 55 and 50 male and female patients, respectively). The mean age was 27.4±6.4 years (ranging from 18to 50years)with a median age of 27 years. The age of the prodromal symptoms onset was 19.1 ±7.4 years and the age at schizophrenia onset was 23.9±7.2 years. The disease durations from the beginning of the prodromal symptoms onset and from the onset of the active phase were 8.6±7.1 years and 3.7±4.6 years, respectively.

## RESULTS

The proportion of seropositive patients in the sample was 16.2% (17 out of 105 cases). When comparing Toxoplasma-seropositive or seronegative schizophrenia patients, no statistically significant differences were found in parameters such as the mean total PANSS score, mean PANSS-P, PANSS-N or PANSS-G scores, although there was a general trend towards a higher mean total score for all subscales and the total PANSS score among patients who were seropositive (Table 1).

**Table 1:** Results of comparison of PANSS items in schizophrenia patients depending on their seropositive (lg+) or seronegative (lg-) status (M±SD)

Item	Ig+ (n=17)	Ig- (n=88)	U	р
Mean PANSS total score (Ʃ)	103.7±23.8	96.0±28.6	735.0	0.266
Mean PANSS-P (Positive Scale) score	26.4±6.1	23.8±8.4	881.0	0.182
Mean PANSS-N (Negative Scale) score	28.2±9.4	24.9±9.8	837.5	0.262
Mean PANSS-G (General Psychopathology Scale) score	49.3±11.7	47.0±13.2	739.5	0.525

For the majority of PANSS items, differences were also statistically insignificant, except for two items on the PANSS-G scale—G5 and G6 (Table 2). The total score for "G5. Mannerisms and posturing item" in seropositive patients was statistically significantly higher at 3.5 points versus 2.1 points (p=0.002) and "G6. Depression" total score in seropositive patients was statistically significantly lower at 1.4 points versus 2.4 (p=0.025).

Given the statistical difference for the item G5 (mannerisms and posturing), an additional analysis of clinical data was conducted. The differences between the groups of seropositive and seronegative patients were analysed by categorical variables, which also included descriptions of motor phenomena observed in schizophrenia (primarily catatonia). In accordance with the ICD-10 criteria for schizophrenia, seropositive patients demonstrated a statistically significantly higher rate of mutism (23.5% versus 3.4%, χ^2^=9.27, p=0.013). However, the frequency of other ICD-10 defined catatonic symptoms was similar in both groups. Based on E. BleuleTs criteria (1911) [11], a statistically significant difference was observed for all catatonic symptoms (52.9% versus 28.4%, χ^2^=3.916, p = 0.048).

## DISCUSSION

The proportion of seropositive patients in the sample of schizophrenia patients established in this study (16.2%) is consistent with the lower values of the range given in the literature of 16.2-46% [5,10] and considerably lower than that obtained in one of the Russian studies, also carried out on the population of patients with schizophrenia in the Moscow region (40%) [7]. This may be partly due to the younger age of patients in this study (mean and median values are 27 years); many were undergoing diagnostic evaluation for their first psychotic episode.

Comparing Toxoplasma-seropositive and seronegative schizophrenia patients, no statistically significant differences were found in parameters such as the mean total PANSS score, mean PANSS-P, PANSS-N or PANSS-G scores. In this respect, our data are consistent with the results obtained by F. Dickerson et al. [5] who did not reveal any differences in the total PANSS score and by D. Holub et al. [9] who also found no differences in the PANSS-N or PANSS-G scores. However, D. Holub et al. [9] and H.L. Wang et al. [10] showed a higher PANSS-P in seropositive patients, which differs from our findings. In our study, although the total PANSS-P scores were higher in seropositive patients, the differences did not reach statistical significance. Moreover, in the study by H.L. Wang et al. [10], seropositive patients were found to have significantly lower PANSS-N scores, which was not observed in our study or by other authors [5,9].

None of the studies showed data on individual PANSS items, which could underlie the differences in question and none of them demonstrated data on individual schizophrenia symptoms assessed using other methods.

The statistically significant differences in respect of the G5 item "Mannerisms and posturing" observed in our study indicate that seropositive patients had more severe catatonic motor symptoms (clumsiness, unnatural movements, discoordination, bizarre and fixed postures, stiffening, habits and stereotypes), which were registered according to the PANSS instructions by physicians who were observing patients during the interview (or based on information obtained from personnel or relatives) [12,13]. The data indicating the association of toxoplasmosis seropositive status and catatonic symptoms were supported by the evidence that seropositive patients were statistically significantly more likely to have mutism (according to the ICD-10 criteria) and all groups of catatonic symptoms according to E. Bleuler's criteria [11].

This association of seropositive toxoplasmosis status and catatonic phenomena in schizophrenia patients may be due to a mild neurological deficit that develops as a result of the neutropic effect of the infection and mimics and/or amplifies motor symptoms associated with schizophrenia. It should be noted that this assumption is consistent with the modern concept of catatonia in the Diagnostic and Statistical Manual of Mental Disorders (DSM-5) and International Classification of Diseases 11th Revision (ICD-11) as a syndrome, which can be seen in a wide range of diseases, both those of the schizophrenic spectrum and organic CNS disorders.

Some studies of organic psychoses in patients with Toxoplasma infection, both congenital and acquired, can be considered as clinical observations indirectly confirming this hypothesis [14,15]. The aforementioned studies provide psychopathological characteristics of schizophreniform disorders associated with toxoplasmosis. Some of them are classified as oneiroid-catatonic conditions (delusional interpretation oneiroids) with mild cloudiness of consciousness. According to the authors, these psychoses could be initially considered hallucinatory-paranoid states [15] with a phenomenon of psychic automatism and a delusional misidentification syndrome or Capgras delusion, as well as religious or love delusions [14]. More severe disorders of consciousness in such patients were associated with catatonic symptoms (substupor or stereotypic arousal). However, in some cases the investigators also observed (as the disorders of consciousness became less pronounced) the phenomenon of a lucid catatonia syndrome. The investigators noted that gradually progressive toxoplasmosis was associated with alterations of exacerbations in the form of substupor states and psychomotor excitement, which was the reason why some patients were initially diagnosed with catatonic schizophrenia [14].

**Table 2:** Comparison of mean PANSS scores depending on seropositive (lg+) or seronegative (lg-) status of patients (M±SD)

Items	IgG+ (n=17), Mean score	IgG- (n=88), Mean score	U	р
P1. Delusions	5.41±1.50	4.52±1.89	540.50	0.064
Р2. Conceptual disorganization	4.71±1.40	3.95±1.71	564.00	0.116
Р3. Hallucinatory behaviour	3.24±2.49	3.28±2.23	723.00	0.820
Р4. Excitement	3.12±1.76	2.75±1.67	655.00	0.403
Р5. Grandiosity	2.53±1.55	2.45±1.81	704.50	0.683
Р6. Suspiciousness/persecution	4.59±1.23	4.11±1.38	583.50	0.160
Р7. Hostility	2.76±1.60	2.60±1.81	697.00	0.642
N1. Blunted affect	4.06±1.68	3.63±1.67	651.00	0.386
N2. Emotional withdrawal	4.29±1.40	3.60±1.52	549.00	0.087
NЗ. Poor rapport	3.88±1.45	3.20±1.63	585.00	0.164
N4. Passive/apathetic social withdrawal	3.94±1.68	3.84±1.60	721.50	0.813
N5. Difficulty in abstract thinking	4.29±1.57	3.57±1.64	561.50	0.111
N6. Lack of spontaneity and flow of conversation	3.71±1.72	3.50±1.71	694.50	0.637
N7. Stereotyped thinking	4.00±1.54	3.53±1.72	618.50	0.278
G1. Somatic concern	2.06±1.56	1.90±1.40	695.00	0.601
G2. Anxiety	3.18±1.24	3.28±1.38	674.50	0.507
GЗ. Guilt feelings	1.53±1.33	1.66±1.29	691.00	0.513
G4. Tension	2.76±1.30	3.09±1.46	650.50	0.382
G5. Mannerisms and posturing	3.53±1.74	2.11±1.68	389.50	0.001
G6. Depression	1.41±0.94	2.41±1.76	509.50	0.023
G7. Motor retardation	2.35±1.62	1.94±1.46	644.50	0.305
G8. Uncooperativeness	2.71±1.69	2.60±1.65	729.00	0.863
G9. Unusual thought content	4.00±1.17	3.48±1.39	593.00	0.164
G10. Disorientation	1.88±1.17	1.65±1.05	671.50	0.434
G11. Poor attention	4.18±1.63	3.61±1.64	593.00	0.191
G12. Lack of judgement and insight	4.88±1.65	4.37±1.71	606.50	0.234
G13. Disturbance of volition	4.31±1.25	3.74±1.55	561.00	0.184
G14. Poor impulse control	2.88±1.69	2.91±1.71	745.50	0.982
G15. Preoccupation	4.47±1.46	4.06±1.65	630.00	0.294
G16. Active social avoidance	4.00±1.37	3.94±1.50	726.50	0.848

Some hypotheses suggested by other authors may be considered biological grounds that are consistent with our working hypothesis [10,16-18]. Thus, the heterogeneity of psychopathological and neurological symptoms associated with Toxoplasma invasion may be associated with patterns of distribution of parasite cysts in the host brain. Predominant topical localization of cysts in dopaminergic structures in some cases might cause the development of schizophrenic symptoms or amplification of certain schizophrenic phenomena, e.g., catatonic manifestations, which are considered as a trans-nosological construct in modern research. Some authors believe that the mechanism underlying this association may be due to the ability of *T. gondii* to increase dopaminergic activity [19,20] or its effects on tryptophan metabolism in the corresponding structures [21], which requires verification in further morphological and neuroimaging studies.

The discussion of the association between depression-related symptoms in seropositive patients is beyond the scope of this article. However, we may stipulate that this is consistent with the findings of a clinical and epidemiological study by A.B. Smulevich et al. [22] who investigated the association of depressive and other schizophrenia symptoms and demonstrated a negative correlation between the rate of depressive and catatonic symptoms.

## CONCLUSION

Our results may be relevant for further research on the nature of the association of seropositive toxoplasmosis status with clinical manifestations of schizophrenia and its underlying mechanisms.

Given the inconsistency of available published data regarding the association of toxoplasmosis seropositive status with individual dimensions on the PANSS scale (primarily positive and negative items), it is probably too early to draw unambiguous conclusions about this relationship. Future research in larger samples and/ or a meta-analysis of available publications is needed.

However, we have not been able to find information on the association of seropositive status with catatonic symptoms in schizophrenia in the available publications and it is probably discussed for the first time, which determines the novelty of this study. Our data on the association with catatonic symptoms need to be replicated and further confirmed using biological methods, taking into account the clinical characteristics of schizophrenia. The obtained data can be used for differential diagnosis of endogenous and organic psychoses and therefore, for differential treatment approaches taking into account the possible role of toxoplasmosis in schizophrenia, as well as its potentially modifying effects on the clinical manifestations of schizophrenia.

## Author Contributions

Dmitry V. Romanov, Alexey Yu. Brazhnikov, George P. Kostyuk, Denis S. Andreyuk, Dmitry B. Goncharov: development and approval of the study design; Denis S. Andreyuk, Natalia V. Zakharova, Lidia V. Brawe: obtaining clinical data for analysis, preliminary analysis of the obtained clinical data; Vasilisa A. Kovaleva, Evgenia V. Abbazova, Dmitry B. Goncharov, Irina V. Titova, Elvira A. Domonova: obtaining immunological data for analysis, preliminary analysis of the obtained immunological data; Alexey Yu. Brazhnikov, Dmitry V. Romanov: statistical data analysis, summary and final analysis of the obtained data; Dmitry V. Romanov, Dmitry B. Goncharov: writing the text of the manuscript; Vasilisa A. Kovaleva, Dmitry V. Romanov: review of publications relevant to the article.

## Funding

The study was conducted without the sponsor's support.

## Conflict of Interest

The authors declare no conflicts of interest.

## Informed Consent

All patients signed the informed consent form for participation in the study and testing of biological samples.
